# Photosynthetic Regulation Under Salt Stress and Salt-Tolerance Mechanism of Sweet Sorghum

**DOI:** 10.3389/fpls.2019.01722

**Published:** 2020-01-15

**Authors:** Zhen Yang, Jin-Lu Li, Lu-Ning Liu, Qi Xie, Na Sui

**Affiliations:** ^1^ Shandong Provincial Key Laboratory of Plant Stress, College of Life Sciences, Shandong Normal University, Jinan, China; ^2^ Shandong Provincial Key Laboratory of Microbial Engineering, School of Biological Engineering, Qilu University of Technology (Shandong Academy of Sciences), Jinan, China; ^3^ Institute of Integrative Biology, University of Liverpool, Liverpool, United Kingdom; ^4^ College of Marine Life Sciences, Ocean University of China, Qingdao, China; ^5^ State Key Laboratory of Plant Genomics, Institute of Genetics and Developmental Biology, The Innovative Academy of Seed Design, China University of Chinese Academy of Sciences, Beijing, China

**Keywords:** sweet sorghum, salt-tolerance mechanism, Na^+^ exclusion, photosynthesis, sugar content

## Abstract

Sweet sorghum is a C4 crop with the characteristic of fast-growth and high-yields. It is a good source for food, feed, fiber, and fuel. On saline land, sweet sorghum can not only survive, but increase its sugar content. Therefore, it is regarded as a potential source for identifying salt-related genes. Here, we review the physiological and biochemical responses of sweet sorghum to salt stress, such as photosynthesis, sucrose synthesis, hormonal regulation, and ion homeostasis, as well as their potential salt-resistance mechanisms. The major advantages of salt-tolerant sweet sorghum include: 1) improving the Na^+^ exclusion ability to maintain ion homeostasis in roots under salt-stress conditions, which ensures a relatively low Na^+^ concentration in shoots; 2) maintaining a high sugar content in shoots under salt-stress conditions, by protecting the structures of photosystems, enhancing photosynthetic performance and sucrose synthetase activity, as well as inhibiting sucrose degradation. To study the regulatory mechanism of such genes will provide opportunities for increasing the salt tolerance of sweet sorghum by breeding and genetic engineering.

## Introduction

Soil salinization is a compelling environmental problem worldwide (Landi et al., 2017). Over ~6% of the world’s lands are saline and ~20% of irrigated lands are currently salt-affected (Song and Wang, 2015; Yuan et al., 2016a). Salt stress is an important factor determining plant growth and yields (Mahajan and Tuteja, 2005; Deng et al., 2015), by substantially affecting key biological processes, such as photosynthesis (Feng et al., 2014), energy metabolism (Song et al., 2016), protein synthesis, and lipid metabolism (Sui et al., 2010; Sui and Han, 2014; Sui et al., 2018). A limited number of crops can survive in saline land, which can lead to desertification (Flowers and Colmer, 2010). Another two limiting factors for the development of the economy in many developing countries are the shortage of cultivated land and energy crisis (IEA, 2019). At present, these developing countries are facing the problem of “large population and less land”. As a consequence, the development of biomass energy and feed crops is restricted by the reduction of cultivated land resources. If energy crops and feed crops with high salt tolerance can be widely planted on saline soils, it is of strategical importance to provide solutions for addressing the challenges of energy and food security.

Plants have evolved various adaptive mechanisms in response to salt stress, leading to survival in saline land. These mechanisms occur at multiple levels, the molecular level (Yuan et al., 2016b) and the physiological and biochemical levels (Guo et al., 2012; Kong et al., 2016; Leng et al., 2018). At the molecular level, many genes related to salt-stress responses are differentially expressed after plants sense the external salt-stress signals (Zhu, 2001). For example, OsCCC1 plays a significant role in K+ and Cl− homeostasis (Kong et al., 2011), overexpression of SsHKT1;1 (Shao et al., 2014), AtZFP1 (Han et al., 2014), SsCHLAPXs (Pang et al., 2011), and PcAPX (Cao et al., 2017) enhances plant salt tolerance. The differential expressions of these genes then drive the salt-stress responses at the physiological and biochemical levels (Cui et al., 2018). The first step is to block Na^+^ transport to shoots through apoplastic barriers, including Casparian bands and suberin lamellae (Krishnamurthy et al., 2011). The second step is to dilute Na^+^ in the cytoplasm through Na^+^ compartmentalization by transporting Na^+^ into the vacuole (Blumwald, 2000). The third step is to exclude Na^+^ outside using a Na^+^/H^+^ antiporter in the plasma membrane. The transport of Na^+^ from roots to leaves can also be restricted by a high-affinity K^+^ transporter (HKT) (Byrt et al., 2007; Byrt et al., 2014).

Sweet sorghum [*Sorghum bicolor* (L.) Moench], which was generated from grain sorghum, has been widely cultivated for animal feed production in tropical and subtropical regions and, in particular, has a large planting area in Asia, Africa, and many developing countries (Antonopoulou et al., 2008). India is the largest sorghum grower in the world, with 7.5 million hectares of lands for growing sweet sorghum, followed by Nigeria (7.6 million hectares) and Sudan (6.6 million hectares) (Rao et al., 2013). In America, about 2.6 million hectares were devoted to sorghum for grain and 153,780 hectares were used for sorghum silage (Getachew et al., 2016). In China, about 100,000 hectares of lands were used to plant sweet sorghum. Sweet sorghum has the characteristics of high photosynthetic efficiency (Guo et al., 2018a), high fermentable sugar content in stalks, and high salt tolerance (Vasilakoglou et al., 2011; Sui et al., 2015). As a C4 crop, it has the ability to produce high biomass under stress conditions. Its high nitrogen-use efficiency enables it to produce two to three times more biomass per harvest than the more commonly used silage crop such as maize (Xie and Xu, 2019). Compared with the original feed crops, sweet sorghum presents the advantages of possessing high temperature and drought tolerance levels, high yields, low soil fertility requirements, and good palatability and digestibility (Chen L. et al., 2018). It has been reported that the protein content in sweet sorghum leaves is up to 22% (Ferraris and Charles-Edwards, 1986). A meta-analysis of feeding experiments found that cows fed with sweet sorghum silage could produce more milk (1.64 kg/day/cow) with an improved quality compared with those fed with maize silage (Sánchez-Duarte et al., 2018). Additionally, most sorghum accumulates high amounts of tannins in the seeds. It has been shown that tannins can promote milk production, lambing percentage, and growth of wool, as well as reducing the risk of rumen bloat (Patra and Saxena, 2011). In an *in vitro* experiment, the supplement of 15 mg tannins in 500 mg of total oven-dried guinea grass reduced methane (CH4) production by 47% (Tan et al., 2011). These findings imply another important advantage of using sweet sorghum silage over other crops: lower methane production and lower damage to the atmosphere. Salt-tolerant sweet sorghum species have stable or increased Brix scores in their stems under salt-stress conditions (Sui et al., 2015). However, salt-sensitive sweet sorghum species often exhibit the decreased net photosynthetic rates and comprehensive utilization degrees of Brix. Salt resistance of sweet sorghum can be improved by the pivotal process of salt exclusion, which induces the accumulation of Na^+^ in the vacuoles of root parenchymata and limits Na^+^ transportation into shoots (Yang et al., 2018).

This review will focus on the physiological and biochemical responses of sweet sorghum to salt stress and the salt-tolerance mechanisms, and discuss how this crop plant can be used to improve saline land.

## Physiological and Biochemical Responses of Metabolic Processes to Salt Stress

Many important metabolic processes are affected by salt stress at the physiological and biochemical levels. Generally, photosynthesis and growth rate of most plants would be inhibited by salt stress. The regulation of these metabolic processes in response to salt stress could modulate the ability of salt tolerance among sweet sorghum accessions.

### Photosynthesis

**Figure 1:**
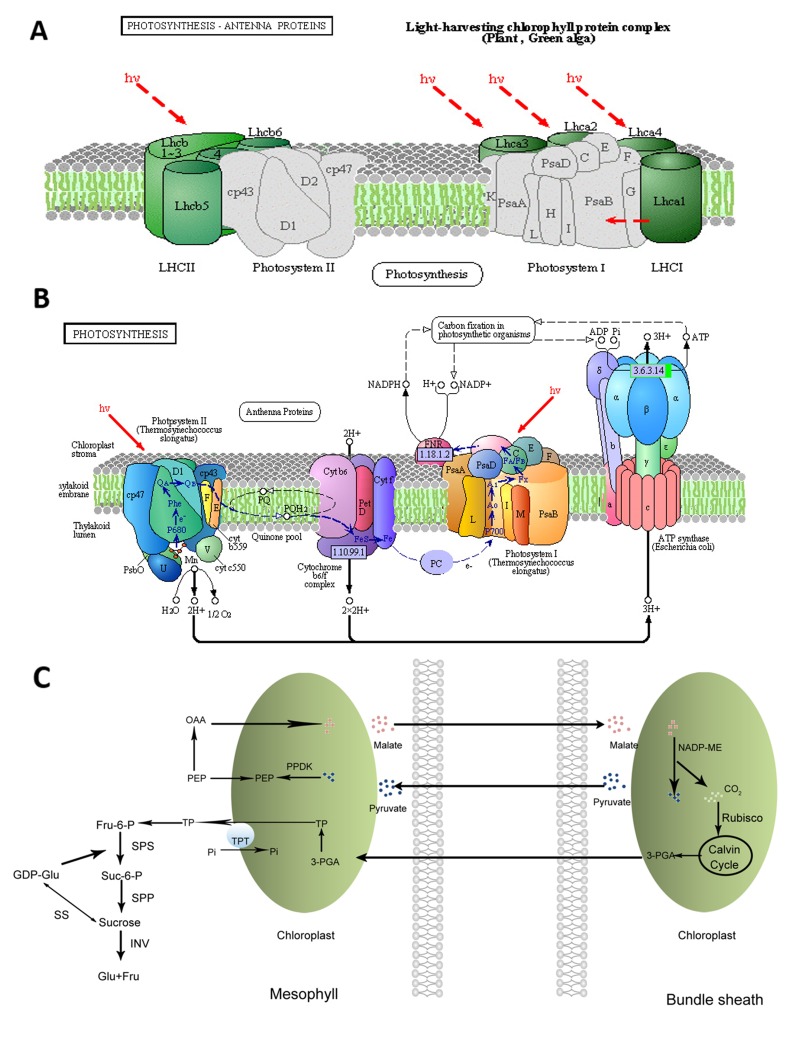
Visualization of pathways related to the accumulation of sucrose in sweet sorghum under salt stress (Repaint refers to Sui et al., 2015). **(A)**: Photosynthetic antenna systems and the light-harvesting process; **(B)**: photosynthetic pathway (Sui et al., 2015); **(C)**: pathway of the carbon fixation in photosynthetic organisms and sucrose biosynthesis.

Photosynthesis is the most important process occurs in the chloroplasts of higher plants. Solar energy can be converted into chemical energy by plants through photosynthesis. Photosynthesis includes light and dark reactions, each of which consists of a series of redox reactions related to energy capture and conversion (Liu, 2016). At the initial step of photosynthesis, light energy is captured by the peripheral light-harvesting complexes, which contain most of the chlorophyll and carotenoid pigments and are peripherally associated with photosystems (PSs) I and II (**Figure 1A**) (Galka et al., 2012). For many plants such as tomato, Zygophyllum xanthoxylum, and *Phaseolus vulgaris*, salt stress could affect the chloroplast structures and decrease the chlorophyll content resulting in the reduced photosynthetic rate (Sudhir and Murthy, 2004; Wydrzynski, 2008; Ma et al., 2012).

The decrease in photosynthesis induced by salt stress could be relevant to the stomatal and/or nonstomatal factors in soybean seedlings (Jiao et al., 2017). Salt stress has direct and indirect effects on the chlorophyll content and photosynthetic efficiency of plants. The direct effects are achieved by regulating the activity and expression levels of enzymes involved in chlorophyll biosynthesis and photosynthesis (**Figure 1B**). The indirect effects are achieved by specific regulating pathways such as antioxidant enzyme systems. It has been reported that the maximum quantum yield of photosystem II (PSII; F_v_/F_m_), photochemical quenching coefficient (qP), and electron transport rate (ETR) substantially decreased, whereas non-photochemical quenching (qN) increased in sorghum under saline conditions (Netondo et al., 2004). In poplar (*Populus* spp.), salt stress could inhibit F_v_/F_m_ due to the salt-induced increase of the minimal fluorescence (F_o_) and the notable decline of the maximal fluorescence (F_m_). It could also result in the decrease of qP but could greatly elevate the coefficient of nonphotochemical qN in the light-adapted state (Wang et al., 2007). In contrast, F_v_/F_m_ was almost not affected by salt stress in rice, whereas qN increased in sensitive cultivars with the increasing salt stress (Dionisio-Sese and Tobita, 2000).

**Figure 2:**
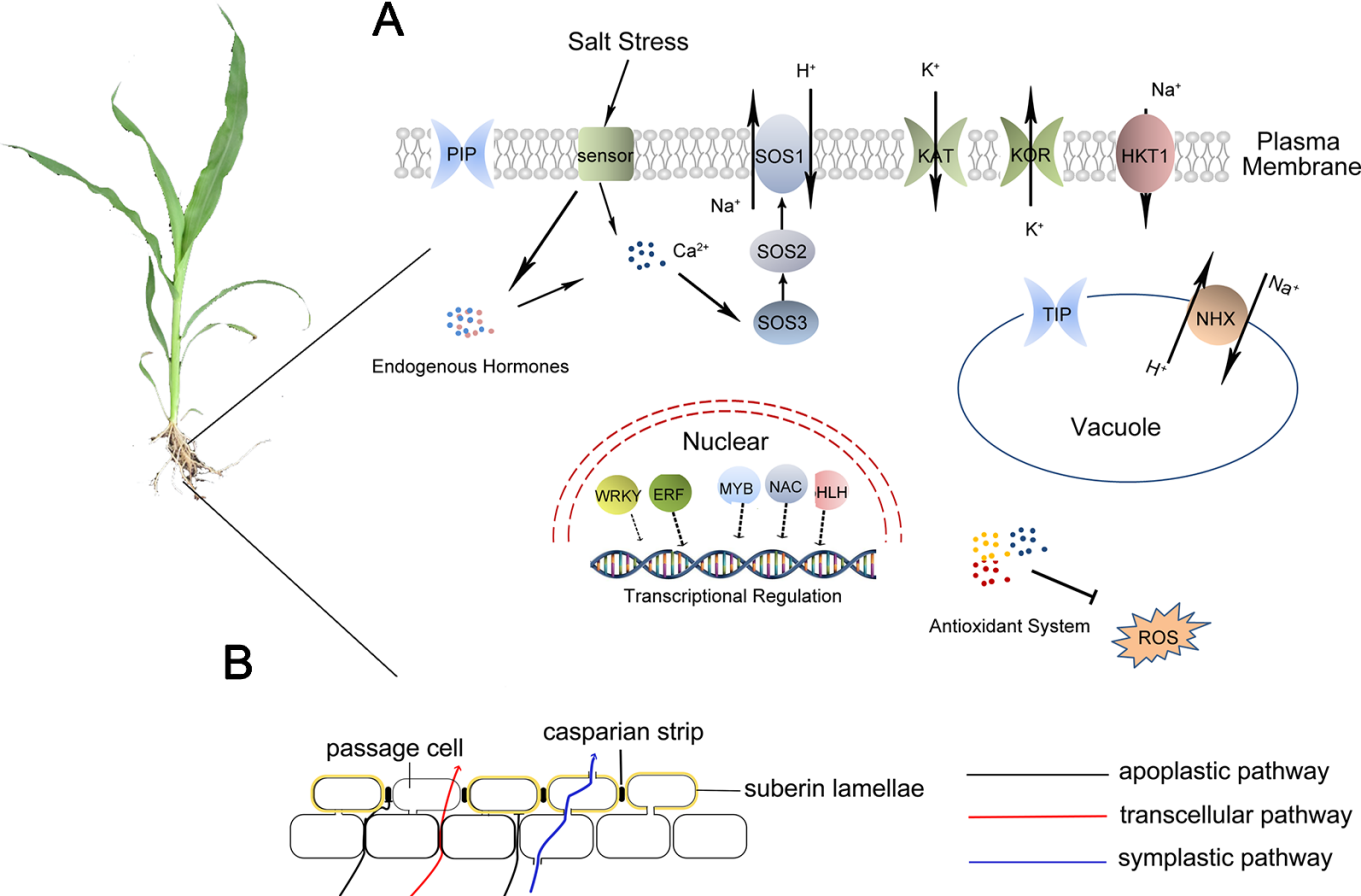
Possible roles of salt resistance in sweet sorghum. **(A)**: Cells of sweet sorghum firstly sense Na+ by an unknown sensor and change the content of intracellular hormones and Ca^2+^. The expression of some transcription factors, such as NAC, bHLH, MYB, is also initiated. These transcription factors then activate the expression of genes encoding proteins related to salt stress response such as SOS1, HKT1, NHX, ROS scavenging proteins, aquaporins, and some ion channels. **(B)**: Physical barrier effect of root apoplastic barriers can block the apoplastic transpiration bypass flow of water and solutes.

It was also reported that salt stress can affect water potential, stomatal conductance, and sugar accumulation to induce inhibition and ion toxicity (Feng et al., 2014). In salt-sensitive sweet sorghum, salt stress was revealed to significantly decrease the net photosynthetic rate, PSII photochemical efficiency, stomatal conductance, and intercellular CO_2_ concentration (Sui et al., 2015). In contrast, salt-tolerant sweet sorghum genotypes can still exploit certain mechanisms to minimize these responses throuth apoplastic barriers (**Figure 2B**). The mechanism of salt-tolerant sweet sorghum for maintaining high photosynthetic efficiency under salt stress may be related to the contributions of several pathways, such as maintaining the stability of the photosynthetic system and enhancing the efficiency of CO_2_ fixation. In the salt-tolerance and salt-sensitive genotypes, F_v_/F_m_ and the actual PSII efficiency (Φ_PSII_) were reduced with increasing NaCl concentration, and the decrease was more significant in Roma which is sensitive to salt stress. The chlorophyll content in M-81E sweet sorghum was not altered greatly by 50 mM NaCl, whereas it decreased in Roma (Sui et al., 2015). No significant changes in the photosynthetic rate, stomatal conductance, and intercellular CO_2_ concentration were observed in M-81E under salt stress. However, these photosynthesis-related indicators in Roma were significantly influenced by salt stress. Additionally, many genes mapped to the photosynthesis pathway showed different expressions in Roma and all of them were down-regulated under salt stress (Sui et al., 2015). Most of these genes are related to the photosystem complexes, the connection between photosystems and light-harvesting proteins, and electron transport chain. Only a few genes in M-81E mapped to the photosynthesis pathway were affected by salt stress, and 2 genes related to the stable assembly of the oxygen-evolving complex and ATP synthase were up-regulated under salt stress (Sui et al., 2015). These results suggest that salt stress could affect the assembly of photosystems and reduce the efficiency of electron transport, resulting in the decrease of ATP and NADPH in plants under salt stress. These effects are particularly critical in salt-sensitive species, whereas salt-tolerant species can protect the formation of photosynthetic assemblies by increasing the expression of particular genes.

Sweet sorghum possesses the C4 pathway of carbon fixation. CO_2_ is fixed initially in the mesophyll cells by phosphoenolpyruvate carboxylase (PEPC) to form malic acid. Malic acid can then diffuse into bundle sheath cells, where they are decarboxylated, and the released CO_2_ is then fixed by Rubisco (Hatch, 1987), the key enzyme in photosynthetic CO_2_ assimilation (Whitney et al., 2011). The phosphorylation of PEPC depends on the interplay of phosphoenolpyruvate carboxylase kinase (PEPC-k) and a 2A-type protein phosphatase (Carter et al., 1990; Vidal and Chollet, 1997). It has been reported that the PEPC-k content in leaves of sorghum could be induced by salt stress under both light and dark conditions, which contributes to the improvement of carbon fixation efficiency (Echevaria et al., 2001; García-Mauriño et al., 2003). NADP-Malate dehydrogenase (NADP-ME) is a critical enzyme of the C4 pathway, which catalyzes the oxidative decarboxylation of malic acid to provide CO_2_ to Rubisco. It has been shown that the expression of the gene encoding NADP-ME could be activated by salt stress (Sun et al., 2003; Liu et al., 2007). Recently, it has been revealed that NADP-ME in sweet sorghum is associated with salt tolerance. NADP-ME in M-81E sweet sorghum was activated by salt stress and overexpression of SbNADP-ME increased the photosynthetic capacity in Arabidopsis under salt stress (Guo et al., 2018b). The increase of NADP-ME may lead to an increase of CO_2_ and pyruvate levels, which enhanced the CO_2_ fixation efficiency. Additionally, NADPH can also participate in the reactive oxygen species (ROS) metabolism by providing power (Mittler, 2002). Møller reported that the NADPH-specific glutathione reductase (GR) can use NADPH to catalyze glutathione reduction for scavenging ROS (Møller, 2001).

### Sucrose Synthesis, Metabolism, and Transportation

Sweet sorghum represents an excellent model for fermentative production due to the fact that its stem contains rich fermentable sugars, such as sucrose, fructose, and glucose (Almodares and Hadi, 2009). Sucrose is the main sugar accumulated through photosynthesis, accounting for ~85% of the total content, in the stems of sweet sorghum (Gnansounou et al., 2005; Kühn and Grof, 2010). The flow direction of triose phosphate formed during photosynthesis determines the distribution of photosynthetic products. It remains in the chloroplast stroma for the completion of the Calvin cycle or to be converted to starch or enters the cytoplasm *via* triose phosphate translocator and synthesizes sucrose under the action of a series of enzymes, such as sucrose phosphate synthetase (SPS), sucrose synthetase (SS), and invertase (INV). Sucrose is temporarily stored in the vacuoles or is exported using long-distance transport through the phloem (**Figure 1C**).

Plant organs are generally divided into the source and sink organs (Turgeon, 1989). A source organ possessing photosynthetic activities usually has a net output of light assimilation, which occurs in the mature leaf. Sucrose is the main source of carbon and energy in the epidermal tissue of plants. It is synthesized in the cytoplasm of leaves (source tissues) where it is loaded into the phloem and is transported to sink tissues (Guimaraes et al., 1997; Mccormick et al., 2010). Sucrose can be transported from the phloem to sink tissues through two pathways (Qazi et al., 2012). In sugarcane, the radial transfer of sucrose from the vascular bundles to parenchymal cells of the sucrose-storing internodes follows a symplastic pathway (Rae et al., 2005). While, in sorghum, it follows an apoplastic pathway (Tarpley and Vietor, 2007). Additionally, sucrose transporters (SUTs) are involved in the long-distance transport of sucrose through the phloem, fulfilling the loading and unloading functions. Among these SUTs, SUT1 is highly specific for sucrose transport in monocotyledonous plants (Scofield et al., 2007; Reinders et al., 2010; Slewinski and Robert and Braun, 2010). The expression of SUT1 in the internodes of sweet sorghum was lower than that in grain sorghum, which may reflect the reduced retrieval of sucrose in the phloem and consequently, an enhanced efflux into storage parenchymal cells (Qazi et al., 2012).

Many enzymes, such as SPS, SS, and INV, are tightly involved in sugar metabolism (Turgeon, 1989). SS can catalyze both the synthesis and the decomposition of sucrose (Schäfer et al., 2005; Hoffmann-Thoma et al., 2010). SPS takes part in the synthesis of sucrose phosphate, which can be converted into sucrose by the catalysis of sucrose phosphate phosphatase (Lunn and Macrae, 2003). INV plays a key role in the process of sucrose degradation and mainly catalyzes the decomposition of sucrose to monosaccharides (Lunn and Macrae, 2003).

### Hormones

Hormones play essential roles in the germination, growth, and development of plants (Chen et al., 2013a; Li et al., 2015). Hormones and their signaling pathways are integrated to allow the triggering of appropriate cellular and physiological responses to adapt to stress circumstances (Ryu and Cho, 2015). To cope with abiotic stresses, several plant hormones regulate defense response reactions (Kazuo et al., 2009; Ahmad and Prasad, 2012). Plant hormones play important roles in responses to salt stress (Javid et al., 2011; Zeng et al., 2018). At the physiological and biochemical levels, sweet sorghum triggers an endogenous hormone-related signaling cascade after sensing external salt stress, which provokes downstream salt-stress responses. Under salt-stress conditions, sweet sorghum can restrict the transport of Na^+^ to the aboveground plant parts by salt exclusion and enhance the antioxidant system simultaneously, and resist the secondary oxidative stress caused by salt stress (Sui et al., 2015; Yang et al, 2018). These protective mechanisms allow sweet sorghum to maintain high photosynthetic efficiency under salt-stress conditions. Maintenance of high photosynthetic performance, up-regulation of genes functioning in sucrose synthesis (SPS and SS encoding genes), and down-regulation of genes related to sucrose degradation (INV-encoding genes) triggered by salt stress lead to the accumulation of sucrose of salt-tolerant sweet sorghum lines.

Among the known hormones, abscisic acid (ABA), jasmonate (JA), ethylene, and cytokinin (CK) have been demonstrated to mediate plant defense responses against abiotic stresses (Nakashima and Yamaguchishinozaki, 2013). One of the fastest responses to abiotic stress is the accumulation of ABA, which is a key regulator in the activation of plant cellular adaptation to drought and salt stresses (Chen et al., 2013b). The increased levels of ABA facilitate the binding to its receptor to initiate signal transduction, leading to cellular responses to stresses (Ng et al., 2014; Sah et al., 2016). Additionally, many other salt-stress-related pathways, such as proline accumulation and calcium signaling, are also physiologically linked to the ABA signaling pathway (Yang et al., 2017). CKs play roles in the regulation of plant responses to stress (Ha et al., 2012). Unlike ABA, CKs can play positive or negative roles under stress conditions (Zwack and Rashotte, 2015; Yang et al., 2017). Colebrook et al. (2014) illustrated that the gibberellin levels affect plant growth under several stress conditions. Although hormones are critical in the salt-stress responses of sweet sorghum, the key hormones functioning in the response reactions vary among sweet sorghum genotypes and possess distinct levels of salt tolerance. Yang et al. (2017) reported that in the salt-tolerant inbred line M-81E, ABA may play a key role in the salt-stress response. However, in salt-sensitive inbred lines, JA may act as the key regulatory hormone of salt stress. It is presumably that ABA and JA regulate the growth and development of sweet sorghum in different tissues of sweet sorghum. ABA plays roles in predominantly leaves whereas JA is active mainly in roots (Yang et al., 2017). It has been described that ABA modulates the degradation of PEPC-k in sorghum leaves by increasing kinase activity in the illuminated leaf of the non-stressed plant and decreasing PEPC-k activity in the dark (Monreal et al., 2007). These results indicated that the changes of ABA content in the leaves of salt-tolerant sweet sorghum variants may be related to the maintenance of high photosynthetic efficiency of sweet sorghum under salt stress.

### Maintenance of Ion Homeostasis

Under salt stress, sorghum can operate osmotic regulation by transporting ions into vacuoles or accumulating soluble substances (Lacerda et al., 2003). Sweet sorghum can scavenge ROS through enzymatic and non-enzymatic antioxidant systems, adapting to secondary oxidative stress caused by salt stress (Chai et al., 2010). In addition, sweet sorghum can also accumulate Na^+^ in roots and limit the transportation of Na^+^ up to shoots under salt stress, which is called salt exclusion (Dai et al., 2014), an important salt-tolerance-related process in monocotyledonous crops, including rice, maize, and sweet sorghum. Plants having the characteristics of salt exclusion can accumulate Na^+^ in the vacuoles of root parenchyma and suppress Na^+^ transportation into shoots. Root apoplastic barriers consist of Casparian bands and suberin lamellae. The formation of apoplastic barriers can block the apoplastic transpirational bypass flow of Na^+^ ( Krishnamurthy P et al., 2009; Yang et al., 2018) (**Figure 2**). Additionally, the high-affinity potassium transporter (HKT) plays important roles in retrieving Na^+^ transport from roots to shoots. Salt stress induces the expression of SbHKT1;4 in salt-tolerant inbred sorghum, and it is vital for balancing the ratio of Na^+^/K^+^ to improve plant growth (Wang et al., 2014). The combining effects of apoplastic barriers in roots and Na^+^ transport by HKTs lead to Na^+^ accumulation in roots. The accumulated Na^+^ is finally transported into the vacuoles through NHXs or out of root cells by the Na^+^/H^+^ antiporters in the plasma membrane (Yang et al., 2018) (**Figure 2A**).

### ROS Scavenging

In plants, dynamic balance of the ROS content is essential for plant growth. However, when the ROS accumulation exceeds the tolerance of the cell, it will cause damage to plants by attacking membrane structure or mediating apoptosis (Xue et al., 2013). Salt stress can induce the accumulation of ROS, which could greatly affect plant photosynthesis, metabolism, signal transduction, and other physiological and biochemical processes. The antioxidant system is adopted in plants to scavenge ROS and free radicals to prevent damages caused by ROS (Sui et al., 2015). Antioxidant enzymes serve as important components of the antioxidant system to remove ROS in plants and increase plant resistance, allowing the maintenance of physiological growth and development. For instance, superoxide dismutase is used to scavenge O_2_^−.^ by converting it to H_2_O_2_. Catalase is used to scavenge H_2_O_2_ by catalyzing it to H_2_O and O_2_ (Mittler, 2002). The expression of ROS-associated antioxidant enzymes is commonly induced by salt stress (Evelin et al., 2009; Xue et al., 2013). Sorghum could use these antioxidant enzymes to adapt to salt and drought stresses (Jogeswar et al., 2006).

### Osmotic Stress

The responses of plants to salt stress are characterized by two phases. The first phase is determined by osmotic stress and the second phase is ionic stress (Munns, 1993). Plants are capable of adjusting their water balance in response to salt stress. Plants adapt to osmotic stress mainly by reducing transpiration and accumulating osmotic adjustment substances (Chen M. et al., 2018). It has been reported that sorghum accumulated proline and soluble carbohydrates after NaCl treatment for 20 days (Heidari, 2009). The accumulation of these substances reduced the water potential of sorghum and maintained the water-absorbing capacity under salt stress. The aquaporin proteins play important roles in transportation of water across biological membranes, which is crucial for plants to combat salt stress (Xin et al., 2014). In sweet sorghum, the aquaporin-encoding genes in both salt-tolerant and salt-sensitive lines were up-regulated under salt stress, which may promote water transport efficiency (Yang et al., 2018). Additionally, the stomatal conductance in leaves of sweet sorghum was notably decreased under salt stress, which plays an important role in reducing water loss through transpiration (Sui et al., 2015).

## Responses to Salt Stress At Different Developmental Stages of Sweet Sorghum

Sweet sorghum has high salt tolerance (Vasilakoglou et al., 2011; Sui et al., 2015). However, the salt effects on sweet sorghum vary among species and occur in different developmental stages. Germination is the starting point for the growth and developmental processes for all crops. Therefore, high germination ability is important for crops in saline soils. The inhibition effects of salt stress on plant germination are mainly through two ways. Primarily, salt stress can drastically decrease the osmotic potential of the soil solution to retard water absorption of seeds. Additionally, salt stress can also cause the sodium and/or chloride toxicity to the embryo and alter protein synthesis (Panuccio et al., 2014). Seed germination is a major factor limiting the establishment of plant populations under saline conditions. Salt-tolerant sweet sorghum germplasms can maintain a good germination state under salt stress (Almodares et al., 2007; Patanè et al., 2009; Patanè et al., 2013). However, under salt-stress conditions, the germination of salt-sensitive sweet sorghum germplasms is significantly inhibited. The germination rate, germination index, germination energy, and fresh weights of roots and shoots were all decreased under salt stress, which result in low densities and low yields (Ding et al., 2018).

It has been reported that the increase in the Na^+^ concentration and the decrease in the K^+^ concentration in salt-sensitive sweet sorghum germplasms during salt stress could lead to the decreased levels of several synthesis and metabolism actions, such as photosynthesis and nutrient transport (Sui et al., 2015; Yang et al., 2018), impeding the growth of both roots and shoots (Almodares et al., 2014). These effects appear to be more significant in salt-sensitive sweet sorghum inbred (Yang et al., 2018). These implied that tolerance to salt stress in sweet sorghum may be related to the decrease of Na^+^ and Cl^+^ accumulation and the maintenance of K^+^ level.

Many of the screening experiments on sweet sorghum have been performed under controlled environmental conditions during the early developmental stages, such as germination and seedling stages. The salt tolerance of only a few germplasms under field conditions has been reported (Vasilakoglou et al., 2011; Fan et al., 2013) as the variation in environment (such as alternations in temperature and rainfall) would easily lead to the variation in the growth of sweet sorghum. A study of four sweet sorghum species in Northern Greece has revealed that sorghum grown in intermediate-salt soil (3.2 dS m^−1^) produced more juice and sugar, and have greater crop and ethanol yields than that grown in high-salt soil (6.9 dS m^−1^) (Vasilakoglou et al., 2011). Moreover, the physiological features of sweet sorghum grown in saline soil vary greatly among germplasms. As it has been reported, some physiological features such as seed emergence rate and growth rate of sweet sorghum decreased under salt stress. However, the plant height, stem width, seed emergence rate, and the total leaf area were significantly higher in salt-tolerant sweet sorghum germplasm than that in salt-sensitive counterparts (Ding et al., 2013). In addition, photosynthetic parameters and sugar content were also less affected in salt-tolerant sweet sorghum germplasm than that in salt-sensitive lines (Fan et al., 2013).

## Responses of Metabolic Processes to Salt Stress At the Molecular Level

The responses of metabolic processes to salt stress at the physiological and biochemical levels are closely related to the regulatory expression of some encoding genes. Nowadays, many genes related to salt response in sweet sorghum have been identified and characterized (Buchanan et al., 2005; Sun et al., 2016; Casto et al., 2018). ABA is a key regulator of the activation of plant cellular adaptation to drought and salt stresses. Examination of the differential gene expression in Sorghum after exposure of seedlings to high salinity, osmotic stress, and ABA revealed that 89 genes were induced with one-fold changes bigger than 2 in shoots, and 84 genes were induced with two-fold changes in roots by salt and ABA treatments (Buchanan et al., 2005). Moreover, there is a great overlap in gene expression in response to salt and ABA treatments, which indicated that ABA signaling pathway plays an important role in sorghum in response to salt stress. It has been reported that salt stress can regulate the accumulation of endogenous ABA in sorghum and SbABI5 expression in sorghum roots is a key step in the ABA signaling pathway to improve salt tolerance (Sun et al., 2016). Under salt stress, the ABA synthesis-related gene *Sb04g030640* was up-regulated in the leaves of salt-tolerant sweet sorghum inbred lines and was down-regulated in salt-sensitive inbred lines. However, genes related to ABA metabolism showed the opposite trends in the two inbred lines. Additionally, genes related to the ABA signaling pathway, such as *PYR/PYL* and *PP2C*, also possess different expression patterns. After treatment with NaCl, the PYL8-encoding gene *Sb09g006700* was down-regulated in the roots of salt-sensitive Roma. By contrast, the DEGs-encoding gene *PP2C* was up-regulated in salt-sensitive Roma but was down-regulated in salt-tolerant M-81E (Yang et al., 2017). Genes involved in the synthesis, metabolism, and signal transduction of JA, indole-3-acetic acid, and CK in sweet sorghum were also expressed with various regulatory patterns under salt-stress conditions (Yang et al., 2017).

Root apoplastic barriers, consisting of Casparian bands and suberin lamellae, play pivotal roles in blocking the apoplastic bypass flow of water and ions into the stele and Na^+^ transport into shoots (Pignocchi and Foyer, 2003). Salt stress induces the strengthening of the root apoplastic barriers (Krishnamurthy P et al., 2009). The expression of genes related to apoplastic barriers in sweet sorghum was up-regulated under high salinity conditions (Yang et al., 2018), suggesting that the apoplastic barriers may take part in the salt response of sweet sorghum. HKTs have been proven taking part in retrieving Na^+^ from the xylem vessels thus restricting the transport of Na^+^ from roots to leaves. They are involved in the regulation of Na^+^ and K^+^ transportation and maintenance of Na^+^/K^+^ balance (Byrt et al., 2014). It has been reported that the expression of *SbHKT1;4* was strongly up-regulated in salt-tolerant sorghum, correlating with an optimal balanced Na^+^/K^+^ ratio and enhanced plant growth (Wang et al., 2014). In sweet sorghum, the expression of *SbHKT1;5* was enhanced both in salt-tolerant and salt-sensitive inbred lines after NaCl treatments, and *SbHKT1;5* has a greater expression in the salt-tolerant M-81E (Yang et al., 2018).

In addition, the genes related to ROS scavenging, heat shock proteins, and osmoregulation solutes also showed differential expression in sweet sorghum under NaCl treatment (Yang et al., 2018). With the expression of these genes, the contents of proline, soluble protein, catalase, and peroxidase increased in sweet sorghum under NaCl treatment (Oliveira et al., 2011; Ngara et al., 2012). These accumulations may contribute to the increase of salt-resistance capability of sweet sorghum.

Under salt stress, the genes encoding photosynthetic proteins were also shown to present different expression regulations in distinct sweet sorghum genotypes. The genes encoding Lhca1 and Lhcb1-5 were down-regulated in both salt-tolerant and salt-sensitive sweet sorghum lines under salt stress (Sui et al., 2015). The expression levels of the genes encoding Lcha2-4 and Lchb6 dropped in salt-sensitive Roma under salt stress but did not change in salt-tolerant M-81E. Moreover, the genes *Sb02g002830* and *Sb09g021810* that are related to the stable assembly of the oxygen-evolving complex and ATP synthase were up-regulated, affecting the stability of the photosynthetic apparatus under salt-stress conditions (Sui et al., 2015). As a C4 plant, sweet sorghum uses NADP-malic enzyme, ribulose-bisphosphate carboxylase, phosphoenolpyruvate carboxylase, and pyruvate orthophosphate dikinase as the key enzymes in the dark reaction of photosynthesis. When treated with NaCl, the genes encoding these enzymes in sweet sorghum were down-regulated, indicating that salt stress reduced CO_2_ assimilation. However, genes encoding other enzymes involved in carbon fixation in photosynthetic organisms, such as NADP^+^-malate dehydrogenase, were extremely enhanced by salt stress in only salt-tolerant sweet sorghum genotypes. This could increase the levels of CO_2_, pyruvate, and NADPH, which enhance CO_2_ assimilation (Sui et al., 2015).

So far, the molecular mechanism of comprehensive salt responses in sweet sorghum has been largely unknown. It is necessary to draw great attention on the mechanism of salt tolerance and the functions of salt-tolerant genes/proteins in sorghum.

## Genome-Wide and Genetic Engineering of Sweet Sorghum to Improve Salt Tolerance

The sequencing of the whole sorghum genome was completed in 2008 (Paterson et al., 2008). To date, 820 sorghum protein-encoding sequences have been registered (http://www.gramene.org). The molecular cytogenetic maps have also been completed using multiprobe FISH cocktails technology (Islamfaridi et al., 2002). Zheng et al. (2011) carried out the genome resequencing of sweet sorghum and Chinese sorghum strains using a second-generation high-throughput sequencing platform. In total, 95% of the sequences were mapped to the sorghum genome, and a large number of single-nucleotide polymorphisms were found (Zheng et al., 2011). The completion of the genome sequencing laid the foundation for in-depth study of functional genes in sweet sorghum. Many resistance-related genes, including those encoding key enzymes of some metabolic pathways, and genes important for biological productions have been characterized. Developing an efficient genetic transformation system is pivotal for molecular breeding and understanding the genetic control of plant physiology and development. It is especially necessary for the salt-tolerance breeding of sweet sorghum. Currently, the absence of an efficient sweet sorghum transformation protocol represents a major bottleneck in salt-tolerance genetic engineering (Raghuwanshi and Birch, 2010; Liu and Godwin, 2012).

## Conclusion and Perspectives

To survive under salt stress, plants have developed multiple salt tolerance mechanisms. Briefly, sweet sorghum improves the Na^+^ exclusion ability to ensure a relatively low Na^+^ concentration in shoots. Additionally, salt-tolerant sweet sorghum can maintain a high sugar content in shoots, by protecting the assembly of photosystems, enhancing the biosynthesis of sucrose and inhibiting the degradation. However, many puzzles for understanding the sweet sorghum acclimation to salt stress remain to be elucidated, for example, the regulation of salt-tolerant pathways and their interactions, how to apply the theoretical research outputs to field for crop production. To address these puzzles, more advanced and in-depth studies are required. In the future, some techniques such as gene editing systems may be used to address these questions. It will promote biotechnological applications and molecular breeding of salt-tolerant crops, which can increase the usage of saline land and crop production.

## Ethics Statement

Written informed consent was obtained from the individual(s) for the publication of any potentially identifiable images or data included in this article.

## Author Contributions

ZY and J-LL initiated preparation of the manuscript. NS, QX, and L-NL finalized the manuscript. All authors have read and approved the final manuscript.

## Conflict of Interest

The authors declare that the research was conducted in the absence of any commercial or financial relationships that could be construed as a potential conflict of interest.
